# Expression of C-5 sterol desaturase from an edible mushroom in fisson yeast enhances its ethanol and thermotolerance

**DOI:** 10.1371/journal.pone.0173381

**Published:** 2017-03-09

**Authors:** Ayushi Kamthan, Mohan Kamthan, Asis Datta

**Affiliations:** National Institute of Plant Genome Research, New Delhi, India; National Renewable Energy Laboratory, UNITED STATES

## Abstract

Bioethanol is an environment friendly and renewable source of energy produced by the fermentation of agricultural raw material by a variety of microorganisms including yeast. Obtaining yeast strains that are tolerant to stresses like high levels of ethanol and high temperature is highly desirable as it reduces cost and increases yield during bioethanol production. Here, we report that heterologous expression of C-5 Sterol desaturase (FvC5SD)—an ergosterol biosynthesis enzyme from an edible mushroom *Flammulina velutipes* in fission yeast, not only imparts increased thermotolerance but also tolerance towards high ethanol concentration and low pH. This tolerance could be attributed to an increase of ≈1.5 fold in the level of ergosterol and oleic acid (C-18 unsaturated fatty acid) as analysed by gas chromatography- mass spectrometry. FvC5SD is a membrane localized iron binding enzyme that introduces double bond at C-5 position into the Δ7-sterol substrates to yield Δ5, 7- sterols as products. In *F*. *velutipes*, FvC5SD transcript was observed to be upregulated by ≈5 fold under low pH condition and by ≈ 9 folds and ≈5 fold at 40°C and 4°C respectively when compared to normal growth temperature of 23°C. Besides, susceptibility to cell wall inhibiting drugs like Congo red and Calcoflour white was also found to increase in FvC5SD expressing *S*. *pombe* strain. Alteration in membrane sterol and fatty acid composition could also lead to increase in susceptibility to cell wall inhibiting drugs. Thus, this study has immense industrial application and can be employed to ensure competitiveness of fermentation process.

## Introduction

Currently, there is an ever increasing demand of energy to meet the requirements of growing world population and industrialization. Bioethanol is the most commonly used renewable biofuel. Ethanol can be produced from variety of agricultural wastes including starch and lignocelluloses. Ethanol production is based on the process of fermentation carried out by a variety of microorganisms such as fungi, bacteria, and yeasts. In ethanol production, simultaneous saccharification and fermentation (SSF) is considered to be more efficient and advantageous strategy of bioconversion compared to separate hydrolysis and fermentation (SHF) due to low cost, low end product inhibition, high yield and productivity [1,2, 3].

*Saccharomyces cerevisiae* is one of the most commonly used yeast strain for industrial production of bioethanol. However, the fission yeast *Schizosaccharomyces pombe* can also be utilized for large scale production of ethanol. Both the yeast species share superficial similarities, but are significantly diverged from each other [4]. Abubaker et al. (2012) established the role of *S*. *pombe* as a potential fermenting microorganism that can produce ethyl alcohol from molasses [5]. Yeast are exposed to various kind of stresses during ethanol fermentation including osmotic stress (high concentration of sugar substrate), toxic by-product inhibition, high temperature and increased level of ethanol. Among these, increased level of ethanol is one of the major factors limiting bioethanol production [6]. During fermentation, concentration of alcohol keeps on increasing to the levels that can be toxic or lethal to the cells. Ethanol, when present in high concentrations leads to hyperpolarization of phospholipid of the lipid bilayer of cell membranes and organelles, resulting in increased fluidity and reduced integrity [7, 8].

Genetic improvement of yeast to obtain strains that can combat or adapt to extreme conditions of stress, is an important strategy to ensure the competitiveness of a fermentation process. Recently, increased ethanol production was achieved in yeast by expression of AtMed15 of *Arabidopsis* which led to increased flocculation [9]. The resistance of yeast to high temperature and ethanol concentration are desirable characteristics for production of bioethanol [10]. Thus, identifying or generating ethanol tolerant yeast strains could improve the final ethanol concentration and productivity, which in turn save energy on downstream ethanol recovery [11, 12, 3]. Yeast strains that show tolerance to stress imposed by high ethanol concentration are observed to have certain physiological properties which help them to survive such as intracellular accumulation of ergosterol, trehalose and proline [13, 14, 15]. Besides, there are several advantages associated with using thermotolerant yeasts, such as decrease in cost associated with cooling fermentation vats, higher yields in saccharification, and reduced level of bacterial contamination [16, 17].

Ergosterol, one of the main fungal sterols is involved in important cellular functions such as maintaining fluidity, permeability and integrity of the membranes [18]. Ergosterol also plays an important role in the process of endocytosis [19] and homotypic vacuole fusion [20]. One gene essential to ergosterol biosynthesis is ERG3, which encodes the Δ7-Sterol-C5 (6)-desaturase responsible for introducing a double bond at C-5 in the B ring of episterol [21, 22, 23]. Δ7-Sterol-C5(6)-desaturase is membrane bound enzyme that catalyzes introduction of a C-5 double bond into the B ring of Δ7-sterols to yield the corresponding Δ5,7- sterols in mammals [24], yeast [21], and plants [25]. In yeast, ERG3 was found to be a non-essential gene except under heme deficient condition [26]. ERG3 enzyme is a critical target in ergosterol biosynthesis [27] and its expression is directly affected and regulated by mutations in other enzymes of ergosterol biosynthesis pathway [28]. ERG3 genes are involved in the resistance mechanisms of fungi against azole drugs and polyene compounds [29, 30, 31]. Multiple alignments between different C-5 sterol desaturase orthologs in yeasts, filamentous fungi, plants, and humans [32] showed the presence of three conserved histidine rich motifs (H*XXXX*H, H*XX*HH, and H*XX*HH).The conserved histidine-rich motifs containing the putative iron binding domains are assumed to constitute the active site of the enzyme [33] Furthermore, presence of a consensus motif required for retention of transmembrane protein in the endoplasmic reticulum [34] is common to all the homologs.

The cDNA or genes encoding C-5 sterol desaturase have been cloned from several organisms including *Saccharomyces cerevisiae* [29], *Arabidopsis thaliana* [35], *Homo sapiens* [36, 37], *Candida glabrata* [30], alga *Chlamydomonas reinhardtii* [38] and edible mushroom *F*. *velutipes* [32]. **Table 1** depicts the list of C-5sterol desaturases reported from various organisms.

**Table 1:** C-5 sterol desaturase characterized from various organisms.

Organism	Accession number (GenBank)	References
*Saccharomyces cerevisiae*	M62623	[29]
*Flammulina velutipes*	JN696291	[32]
*Candida albicans*	AF069752	[33]
*Arabidopsis thaliana*	X90454	[35]
*Homo sapiens*	AF069469	[37]
*Nicotiana tabacum*	AF081794	[37]
*Chlamydomonas reinhardtii*	{"type":"entrez-protein","attrs":{"term_id":"159486865","text":"XP_001701457"}}XP_001701457	[38]
*Aspergillus fumigatus*	*ERG3A*: AY616449., *ERG3*B: AY616450	[39]
*Tetrahymena thermophila*	FJ940725	[40]
*Schizosaccharomyces pombe*	AB004539	[41]

FvC5SD is a C-5 sterol desaturase isolated from edible mushroom *Flammulina velutipes* [32]. FvC5SD was shown to be an iron binding transmembrane protein observed to accumulate in the microsomal fraction of the fungal mycelia. FvC5SD was observed to be involved in iron uptake and its transcription was shown to be regulated by iron [32].

In order to increase the suitability of *S*. *pombe* strain for bioethanol production, we aimed to generate *S*. *pombe* strain with improved tolerance to various stresses. In this study, we have reported that heterologous expression of FvC5SD in *S*. *pombe* imparts increased thermotolerance as well as tolerance towards high ethanol concentration and low pH. This tolerance could be attributed to an increase of ≈1.5 folds in the level of ergosterol and oleic acid (C-18 unsaturated fatty acid) as analysed by gas chromatography- mass spectrometry. Besides, susceptibility to cell wall inhibiting drugs Congo red and Calcoflour white was also observed to increase in FvC5SD expressing strain. Alteration in membrane sterol and fatty acid composition could also lead to increase in susceptibility to cell wall inhibiting drugs.

## Materials and methods

### Strains and growth conditions

*Flammulina velutipes* (ATCC13547) was maintained on media consisting of 5.0% Dextrose, 1.0% peptone, 0.1% KH_2_PO_4_, 0.05% MgSO_4._7H_2_O and 1% Malt Extract [42] at 23°C for 15–30 days. *Saccharomyces pombe* strain BJ7468 (ura4-D18, leu1-32, and ade6-M216) was grown aerobically at 30°C in either rich media YPD (1% Yeast extract, 2% peptone, 2% dextrose) or SD minimal media (0.67% Yeast nitrogen base without amino acids 2.0% Dextrose, 2.0% agar) or Edinburgh minimal medium (EMM) as described by [43].The *Escherichia coli* DH5α strain (used for DNA manipulation) was grown in LB medium (Sigma Chem. Co., St. Louis, MO, USA) at 37°C. Strains used in the study are listed in **S1 Table**.

### Cloning in yeast expression vector

FvC5SD cDNA (891bp) was PCR amplified using forward primer 5’GCGGCCGCATGGACGTCGTTCTCAACAT3’ and reverse primer 5’CCCGGGCTAAATAATGCATGAATCTACT3’ to add Not I and Sma I restriction enzyme sites at 5’ and 3’ end respectively. FvC5SD cDNA cloned in T- vector was used as a template for PCR. After double digestion with Not I and Sma I, PCR amplicon was cloned in respective restriction sites of *S*. *pombe* expression vector pSLF 173. It has a thiamine repressible full strength nmt1 promoter along with an N terminal triple HA tag (Haemagglutinin tag).

### Northern and southern blot analysis

Total cellular RNA was isolated from 15-day-old *F*. *velutipes* mycelia using Tripure reagent (Roche) according to manufacturer’s instructions. Total RNA was separated on 1.5% agarose-formaldehyde gel and transferred onto a nylon membrane by capillary blotting. Equal loading was confirmed by Ethidium Bromide and Methylene blue staining. Levels of mRNA on autoradiograms were quantified by densitometry scanning using Flour-S-(Bio-Rad) software.

For Southern blot analysis, genomic DNA was isolated by cetyltrimethylammonium bromide (CTAB) method. 10μg of digested DNA was separated on 0.8% agarose gel and Southern blot analysis was performed as described earlier [44]. High stringency post-hybridization washes were performed at 65°C with 2 X SSC, 1% w/ v SDS and with 0.2 X SSC, 1% w/ v SDS. Blots were probed using [γ-P^32^] ATP labeled FvC5SD cDNA or gene as probe.

### Protein extraction from yeast cells

A single yeast colony from streaked agar plates was inoculated in 10 ml of liquid EMM medium at 30°C overnight. 1% of this pre-culture was then inoculated in 100 ml of liquid EMM till O.D_600_ of 0.6 is achieved. Cells were harvested in oakridge tubes and washed twice with sterile water. Fresh weight of the pellet was recorded and four volumes of pre-chilled glass beads and two volumes of protein extraction buffer were added to the pellet. The tubes were vigorously vortexed for about ten cycles (One minute of vortexing with one minute intervals in ice). The tube was centrifuged at 10,000 rpm for 10 minutes and the supernatant was transferred to a fresh 1.5 ml eppendorf tube. Protein concentration was estimated using Bradford assay. Protein was stored at -20°C.

### Western blot analysis

Total protein was resolved on 12.5% SDS PAGE and electro transferred to nitrocellulose membrane. For detecting endogenous FvC5SD from *F*. *velutipes*, peptide based polyclonal antibody raised in rabbit against FvC5sdp (in 1:10,000 dilution) and alkaline phosphatase conjugated anti-rabbit secondary antibody (1:20,000) were used. Anti- HA monoclonal antibody (Roche) was used at dilution of 1:1000 to detect HA tagged FvC5SD in *S*. *pombe*. Protein bands were visualized using Horse radish peroxidase-conjugated (Amersham) secondary antibodies.

### Transformation of *S*. *pombe*

Genetic transformation of *S*. *pombe* strain BJ7468 was carried out by the alkaline cation method [45]. *S*. *pombe* (BJ7468) single colony was inoculated in 20ml YPD medium for preculture. It was allowed to grow at 30°C for 48hrs at 200 rpm. A fresh 200ml pre-warmed YPD medium was inoculated to 1% from preculture. When culture acquired an O.D_600_ of 0.4–0.5, cells were harvested by centrifugation at 5000g for 5 min.at room temperature. Cells were washed twice with sterile water. Pellet was resuspended in 0.5 volume of 0.1M Lithium acetate (pH 4.9). Pellet was washed twice by 0.1M Lithium Acetate and finally resuspended in1/100 ^th^ volume of 0.1 M Lithium acetate so that it should give final density of 1x10^9^ cells / ml (0.5 O.D has approximately 1x10^7^ cells/ ml).To 100μl of competent cells, 4.0μg sheared salmon sperm DNA and 2.0μg plasmid was added, mixed by gently flicking the tube and incubated at RT for 10 min. 500μl of 50% PEG 3350 in 0.1M Lithium Acetate pH 4.9 was added. Reagents were mixed and incubated at 30°C for 45 min. with intermittent tabbing. Transformation mix was heat shocked at 46°C for 25 min., and then it was allowed to cool at RT. Cells were pelleted down by centrifugation at 13000 rpm for 1 min. PEG was removed and cell pellet was suspended in 200μl of sterile water and plated on SD minimal agar plate. Transformants were selected on the basis of uracil prototrophy.

### Visualization of sterol rich plasma membrane domain

Staining of sterols with filipin was carried out as described earlier [46]. Filipin was added directly to the medium at a final concentration of 5 μg/ml and cells were immediately observed using a confocal microscope (Model No. TCS-SP2., Leica, Germany) with the appropriate filters.

### Indirect immunofluorescence staining

For immunolocalization, yeast cell suspension containing about 10^8^ log-phase cells was pelleted down and suspended in phosphate buffer (0.1 M potassium phosphate, pH 6.5). *S*. *pombe* cells were fixed in 3.7% formaldehyde for 40 min. at 30°C. After centrifugation, cell pellet was washed once and then suspended in 1ml phosphate-sorbitol buffer (0.1 M phosphate, pH 6.5; 1.2 M sorbitol).To it, 10 ml of 2-mercaptoethanol (10 mg/ml) and zymolyase 100T (ICN) was added. Cells were incubated at 30°C for about 40 min or until 70%–80% of the cells form spheroplasts. Spheroplasts were washed with Phosphate-Sorbitol Buffer followed by suspending gently in 1.0 ml phosphate-sorbitol buffer. For each sample, 30 μl of resuspended spheroplasts were added to the polylysine coated slides (Sigma). Slide was incubated for 15 min in a humidified chamber at room temperature (RT). Slides were washed with PBST (0.04 M K2HPO4; 0.01 M KH2PO4; 0.15 M NaCl; 0.1% Tween 20; 10 mg/ml BSA; 0.1 g/100 ml sodium azide) in a Coplin jar for 15 min at RT. To each sample well on the slide, 30 μl of a 5μg/ml concentration of mouse HA tag-specific monoclonal antibody (Roche) was added and slides were incubated overnight at RT in a humidified chamber in dark. Slide was washed 4 times with PBST. To each sample well on the slide, 30 μl of a 1:500 dilution of FITC-conjugated secondary antibody (sigma) was added. Slides were incubated at RT for 2–4 hours in a dark, humidified chamber. Slides were washed 4 times with PBST. Slides were air dried at RT in the dark and samples were examined at 63X magnification under a fluorescence microscope fitted with appropriate filters: (Maximum emission wavelength for samples stained with fluorescein-conjugated antibody is 523 nm).

### Spot dilution assay

The overnight EMM + thiamine grown *S*. *pombe* cell suspension was washed twice, 4-fold serially diluted, and 10-μl aliquot of each dilution was spotted on EMM agar plates (supplemented with leucine, and adenine at concentration of 0.2 mg/ ml) with or without thiamine and incubated at 30°C for 4 days. Uracil was added at concentration of 40μg/ml. Congo red (Sigma) and Calcofluor white (Fluorescent Brightener 28, Sigma) were added at the concentration of 50μg/ml and 40μg/ml, respectively. For ethanol stress tolerance studies, cells were spotted on EMM agar medium with different concentrations of ethanol (5% and 10%).Thiamine was added to the concentration of 15μM or 5μg/ml to repress nmt1 promoter.

### Total lipid extraction and GC-MS

Overnight EMM grown yeast cells were lyophilized, crushed into powder followed by transferring to a vial of glass. To it, internal standard i.e.10 ml of 5α-cholest7en-3β-ol (1 mg/ml stock) was added. Extraction of *S*. *pombe* sterol for GC-MS analysis was done by alcoholic KOH method as reported earlier [47]. 3.0 ml of 25% alcoholic KOH solution was added to the powdered cells and mixed by vortexing for 1.0 min. Cell suspension was transferred to a sterile borosilicate glass screwcap tubes and incubated in a water bath at 85°C for 1hr. After cooling the tubes to room temperature, extraction of sterols was performed by adding a mixture of 1ml of sterile distilled water and 3.0 ml of n-heptane followed by vigorous vortex mixing for 3.0 min. After separation, heptane layer was transferred to a glass screw cap tube. The extract was dried by allowing n-heptane to evaporate completely. The extract was derivatized by adding 100 μl of a mixture consisting of 80 μl BFSTA+20 μl TMCS and incubated at 65°C for 1 hrs. 1μl of derivatized sample was used for GC-MS analysis in the split mode on Shimadzu GCMS-QP 2010 plus as mentioned before (Kamthan et al.2012).

### Bioinformatics analysis

*In silico* analysis for detection of cis-regulatory element was performed by TFsitescan [48].

### GenBank accession

The GenBank accession number of nucleotide sequence of FvC5SD and OXDC gene from *F*. *velutipes* are JN696291 and AF200683 respectively.

## Results

### FvC5SD gene organization and promoter analysis

**Fig 1:**
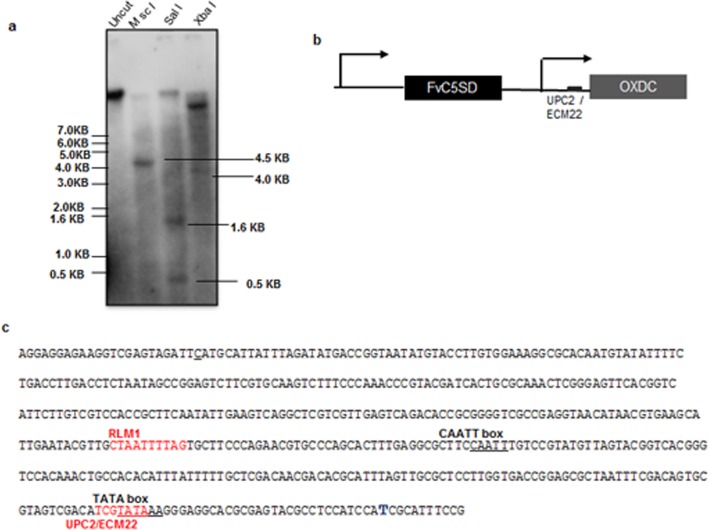
Gene organization of FvC5SD. (a) Southern blot analysis to determine the copy number of FvC5SD in *Flammulina velutipes* genome. Genomic DNA (10μg) was digested with the selected restriction enzymes Msc I, Sal I and Xba I. Blot was hybridized with P^32^ labelled FvC5SD cDNA (891bp) used as probe. Positions of different fragments of 1Kb ladder are shown on left. (b) FvC5SD gene lies upstream to oxalate decarboxylase (OXDC) in the genome. Both the genes have independent promoters. **(**c) OXDC promoter has binding sites of ECM22/ UPC2 transcription factor (-42), which are characteristic of many sterol biosynthesis genes. *In silico* analysis for detection of cis-regulatory element was done by TFsitescan (Ghosh 2000).

C-5 sterol desaturase gene from *F*. *velutipes* was isolated accidentally while trying to clone the 5’ upstream sequence of oxalate decarboxylase (OXDC) gene [49]. A genomic DNA clone was isolated from λgt11 genomic library of *F*. *velutipes* consisting of the nucleotide sequence of the OXDC promoter spanning a region of 0.58 kb. It was analysed that remaining upstream sequence showed significant similarity with the C-5 sterol desaturase gene and was named FvC5SD [32]. Complete FvC5SD gene of *F*. *velutipes* is 1.010 kb in size and is interrupted by two introns based on the presence of matching consensus splice junctions. In FvC5SD gene, splice donar and acceptor sites are typical of eukaryotes i.e. GT and AG at 5’ and 3’ end, respectively ([32] supporting information). Southern blot analysis was done to determine the copy number of FvC5SD in *F*. *velutipes* genome (**Fig 1A**). For this, genomic DNA was digested with three different restriction enzymes that cut the gene at different positions i.e. Msc I, Sal I, and Xba I. High stringency washes were given and blot was probed using γP^32^ labelled 0.89Kb cDNA. FvC5SD was confirmed to be a single copy gene in *F*. *velutipes* genome.

*In silico* analysis of 5’ upstream region of FvC5SD was performed to predict major cis-regulatory elements present in the putative promoter region of the gene (**S2 Table**). Genes involved in sterol biosynthesis generally have sterol regulatory element (SRE) in the promoter which act as binding site for transcription factors like ECM22 and UPC2 [50]. But, no known SRE was detected in FvC5SD promoter. Instead several binding sites for General Control Protein -GCN4 and a DRE (damage response element) were detected at -659 position (**S2 Table**). Mot3 is a zinc finger transcription factor known to play a key role in the regulation of genes required for ergosterol biosynthesis [51]. Three Mot3 binding sites were detected in FvC5SD promoter at -23, -166 and -241. Interestingly, OXDC promoter lying at 3’ UTR of FvC5SD, has binding site for helix-loop- helix transcription factor UPC2 and ECM22 at -42 position further pointing towards some unique kind of co-regulation (**Fig 1B and 1C)**.

### Fv*C5SD* gene is regulated by low pH

**Fig 2:**
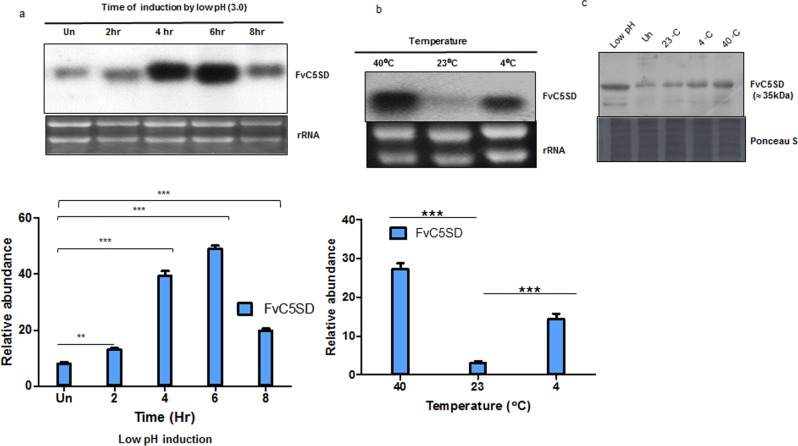
Expression analysis of FvC5SD at different temperature and pH. (a) Northern blot analysis to determine the expression of FvC5SD under low pH condition.15-day-old *F*. *velutipes* mycelia was treated with oxalic acid (to bring down the pH of the medium to 3.0) for desired time points. (b) Northern blot analysis to determine the effect of temperature on FvC5SD expression. *F*. *velutipes* mycelia were grown at 23°C for 15-days followed by exposure to different temperatures (40°C and 4°C) for 6 hours. Upper panel represent autoradiogram of Northern blot analysis done with 20 μg of total RNA and after transfer, blot was probed with P^32^ labelled FvC5SD cDNA. Equal loading was confirmed by ethidium bromide staining of rRNA. Lower panel depicts mRNA levels on autoradiograms quantified by densitometry scanning using flour-S-(Bio-Rad) software. Error bars represents standard error between 3.0 separate biological replicates. Statistical analysis was performed using GraphPad PRISM 5 software. One way analysis of variance (ANNOVA) through Newman Kyle method was used to determine statistical difference between means of three independent groups where *** = P<0.0001 and ** = P<0.001. (c) Western blot analysis of total protein isolated from *F*. *velutipes* mycelia exposed to different conditions was performed using peptide- based polyclonal antibody (raised in rabbit) against FvC5SD. Un, uninduced.

Sequence analysis revealed that the FvC5SD gene is co-oriented with oxalate inducible gene OXDC in *F*. *velutipes* (**Fig 1A**) and each has its independent promoter. However, closely located genes are known to be functionally related and co-regulated, so we performed Northern blot analysis to check whether FvC5SD expression is also regulated by oxalic acid like downstream lying OXDC. Fungal mycelia were induced by adding oxalic acid to the medium to bring down the pH to 3.0. FvC5SD transcript was observed to be upregulated by ≈5 fold after 4–6 hrs upon induction with oxalic acid compared to uninduced control media with pH 5.2. Further reduction in the FvC5SD transcript level was observed after 8hrs (**Fig 2A)**. We also checked the effect on FvC5SD expression when HCl was used to lower the pH of the medium to 3.0. FvC5SD was observed to be upregulated to almost similar levels in presence of HCl (**S1 Fig**). This result shows that FvC5SD expression is regulated by low pH and is not specific to oxalic acid.

### FvC5SD expression is affected by temperature

FvC5SD is an important enzyme of biosynthesis pathway of ergosterol which is known to be an important molecules required for maintaing many properties of fungal membranes.To find out the effect of different temperature on FvC5SD expression, *F*. *velutipes* mycelia was grown at normal growth temperature of 23°C for 15 days followed by exposing to different temperature (4°C and 40°C). After 6.0 hrs of exposure, mycelia were harvested and total RNA was extracted for Northern blot analysis (**Fig 2B**). Expression level of FvC5SD was observed to increase around 9.0 folds at 40°C and around 5 folds at 4°C compared to its normal growth temperature of 23°C. This clearly suggests the role of FvC5SD at extremes of temperature.

Furthermore, Western blot analysis was performed using the total protein isolated from *F*. *velutipes* mycelia exposed to conditions of low pH and extremes of temperature (Fig 2C). Peptide based antibody was used to detect the level of FvC5SD protein. The data correlated well with Northern blot data showing increased expression of FvC5SD protein at low pH of 3.0 as well as extremes of temperature (4°C and 40°C)

### Heterologous expression of FvC5SD in *Saccharomyces pombe*

*S*. *pombe* was used as the system for further characterization of FvC5SD since it showed about 50% identity (highest among yeast) to C-5 sterol desaturase from *S*. *pombe* cDNA of FvC5SD was cloned in *S*. *pombe* expression vector pSLF 173 which has a thiamine repressible full strength nmt1 promoter along with an N terminal triple HA tag (Haemagglutinin tag). After sequencing construct was transformed in BJ7468 strain of *S*. *pombe* by Lithium acetate method and transformants were selected by uracil prototrophy. Western blot analysis (**S2 Fig**) was performed to confirm the expression of HA tagged FvC5SD protein (**≈**38.7kDa) using anti- HA monoclonal antibodies raised in mouse. Triple HA tag was visible in case of control transformed with empty vector.

**Fig 3:**
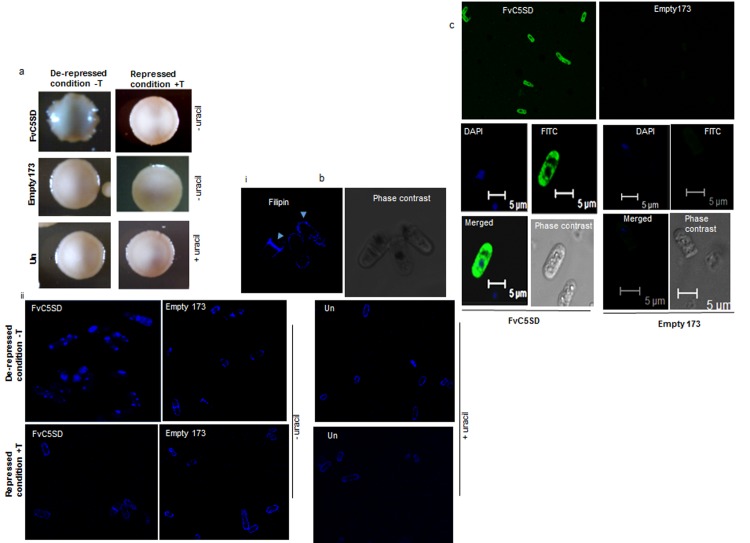
Expression of HA tagged FvC5SD in *S*. *pombe*. FvC5SD was cloned in yeast expression vector pSLF173 (thiamine repressible nmt1 promoter) with triple HA tag at N terminus and transformed in S. *pombe* strain BJ7468. (a) Analysis of colony morphology of *S*. *pombe* expressing FvC5SD/ empty vector or untransformed BJ7468 cells through thiamine repressible nmt1 promoter. (b) i. Typical staining of filipin in tips and septum of *S*. *pombe* cells marked by arrows. ii. Filipin staining of S. *pombe* expressing FvC5SD/ empty vector or untransformed cells. (c) Indirect immunofluorescence staining of *S*. *pombe* expressing HA-FvC5SD/ empty vector was performed with primary antibodies against HA tag followed by incubation with FITC conjugated secondary antibodies (anti-mouse). Confocal microscopy images to show immunolocalization of FvC5SD at 60X magnification. EMM medium with or without thiamine (15μM) was used for repression and re-repression of gene respectively. Uracil (40μg/ml) was added to the EMM medium for growth of untransformed *S*. *pombe* cells (uracil auxotroph). T, thiamine; Un, untransformed.

Morphological characterization of FvC5SD expressing *S*. *pombe* strain was performed in EMM medium in the presence and absence of 15μM thiamine (**Fig 3A**). Colonies formed by *S*. *pombe* expressing FvC5SD in EMM without thiamine medium were slightly irregular in shape. However colonies formed by *S*. *pombe* expressing FvC5SD in repressed condition (EMM+ thiamine medium) were smooth and round similar to empty vector and untransformed control (BJ7468 strain). This difference in morphology could be due to altered sterol composition because of heterologous expression of FvC5SD.

In order to examine the cellular distribution of sterols, *S*. *pombe* cells expressing FvC5SD were treated with the fluorescent probe filipin (**Fig 3B**). Filipin is a polyene antibiotic binds with free 3*β* hydroxysterols to form specific complexes. A strong fluorescence is generated by excitation of filipin at wavelength of 360 nm and an emission maximum at 480 nm. The most prevalent staining pattern in control cells (FvC5SD containing cells under condition of thiamine repression and empty vector/untransformed cells both in presence and absence of thiamine) includes staining at the tips of the cell or additional staining at medial band in elongated cells (**Fig 3Bi**). However, unlike control cells which got stained at cell septum and tips (sterol rich domain), cells expressing FvC5SD, in the absence of thiamine, showed punctuate staining pattern **(Fig 3Bii**). Moreover, overall fluorescent intensity was higher than that of untransformed control cells or that expressing empty pSLF173 vector both in presence and absence of thiamine. It is possible that FvC5SD overexpression is leading to an accumulation of downstream intermediates in the pathway of biosynthesis of ergosterol.

### FvC5SD shows a localization pattern typical of integral membrane proteins

The knowledge of subcellular localization of a protein to determine its localization sites within the cell, helps in elucidating protein functions involved in various cellular processes. FvC5SD is a transmembrane protein that was found to localize mainly in the microsomal protein fraction of fungus *F*. *velutipes* [32]. To study the subcellular localization of FvC5SD expressed in *S*. *pombe*, indirect immunoflorescence microscopy was performed. Fixed spheroplasts of *S*. *pombe* expressing HA-FvC5SD/ empty vector were stained with primary antibodies against HA tag followed by incubation with FITC conjugated secondary antibodies (anti-mouse). *S*. *pombe* cells were counterstained with DAPI (4', 6-diamidino-2-phenylindole) to detect the nuclei. On viewing the samples in confocal microscope, staining of the tagged protein was observed around the nuclear envelope, extending longitudinally to the end of the cell, covering the entire plasma membrane (**Fig 3C**). No signal could be detected in cells expressing empty vector. This result suggests that FvC5SD like most enzymes of sterol biosynthesis pathway, localizes to membranes of *S*. *pombe*.

### FvC5SD expression enhances ethanol tolerance in *S*. *pombe*

**Fig 4:**
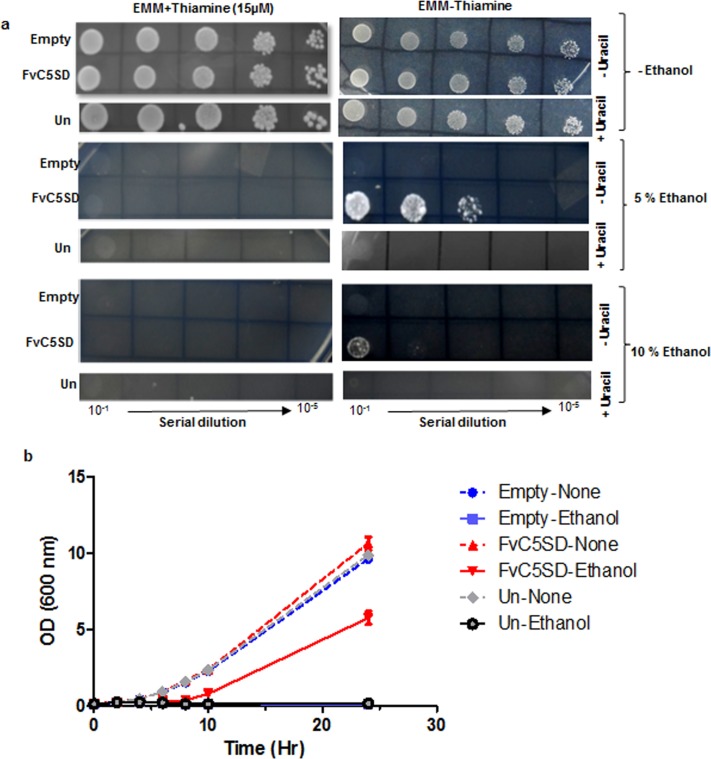
*S*. *pombe* expressing FvC5SD shows ethanol tolerance. (a) Growth of cells on EMM agar presence of different concentrations of ethanol (5% and 10%.). Cells were grown overnight in EMM + thiamine and washed twice with sterile water. Serial dilutions were made and indicated dilutions were spotted on EMM plate in presence or absence of thiamine (15μM) and incubated for 4 days. (b) Growth of cells in liquid EMM media consisting of 5% ethanol in the absence of thiamine. Optical density (O.D) of the cell was monitored by measuring absorbance at 600nm. Uracil (40μg/ml) was added to the EMM medium for growth of untransformed *S*. *pombe* cells. The experiment was performed in three biological replicates.

High concentration of ethanol decreases the permeability of the plasma membrane, causing leakage of essential cofactors and coenzymes leading to inhibitory effect on growth. Ethanol tolerance of an organism depends mainly upon the composition of its cellular membrane consisting of sterols and unsaturated fatty acids [52, 53, 54, 55]. With FvC5SD being a sterol biosynthesis enzyme, upon expression in *S*. *pombe* might lead to changed levels of different sterols in the membrane causing altered membrane properties affecting its tolerance towards ethanol. To determine the effect of FvC5SD expression on ethanol tolerance property, we checked the growth of *S*. *pombe* in presence of higher concentrations of ethanol (5% and 10%). In the absence of thiamine, both untransformed and empty vector strains of *S*. *pombe* showed no growth at either ethanol concentration. S. *pombe* strain expressing FvC5SD was able to sustain growth on EMM agar medium consisting of both 5% and 10% ethanol in the medium without thiamine **(Fig 4A).** Growth in presence of 10% ethanol was slow compared to 5% ethanol. Upon thiamine mediated repression of gene expression, no growth could be observed in any of the strains (FvC5SD/ empty vector/ untransformed control) in presence of ethanol. All the strains (FvC5SD/ empty vector/untransformed control) showed normal growth in the EMM agar control plates without ethanol. However, growth in presence of thiamine was observed to be comparatively faster than in absence of thiamine as reported earlier [56].

Similarly, in the liquid EMM media (without thiamine) consisting of 5% ethanol, FvC5SD expression led to enhanced tolerance to ethanol in *S*. *pombe* as detected by measuring the growth of the cells (optical density at wavelength of 600nm) at different time points upto 24 hours **(Fig 4B)**. In the absence of ethanol, after a brief lag phase, exponential growth was observed in all the *S*. *pombe* strains (FvC5SD/ empty vector/ untransformed control). However, negligible growth was detected in control strains (empty vector/ untransformed) in presence of ethanol. All the experiments were performed in three biological replicates. Hence, it can be concluded that FvC5SD expression leads to enhanced ethanol tolerance in *S*. *pombe*.

### FvC5SD expressing *S*. *pombe* showed enhanced tolerance to low pH, extremes of temperature and increased susceptibility to cell wall inhibitors

**Fig 5:**
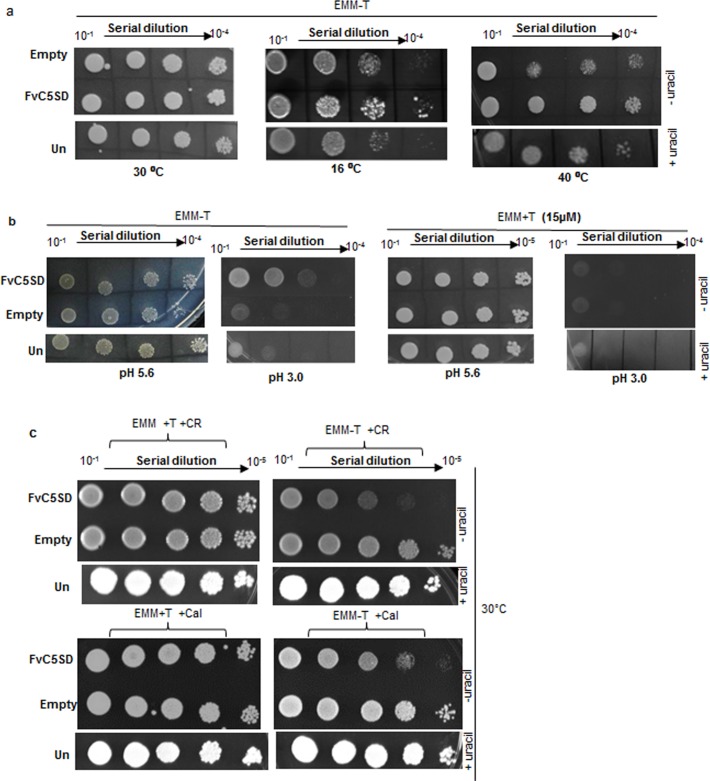
Spot dilution assay to determine tolerance of *S*. *pombe* expressing FvC5SD to pH, temperature and cell wall inhibitors. (a) Growth of cells at different incubation temperature (16°C, 40°C and 30°C). (b) Growth of cells on low pH medium created by adding oxalic acid/ HCl to bring pH of EMM medium to 3.0. (c) Growth of cells in presence of cell wall inhibitors Congo red (50 μg/ml) and Calcoflour White (40 μg/ml). Cells were grown overnight in EMM+ thiamine and washed twice with sterile milliQ water. Serial dilutions were made and indicated dilutions were spotted on EMM plate in presence or absence of thiamine (15 μg/ml) and incubated for 4 days. Uracil (40μg/ml) was added to the EMM medium for growth of untransformed *S*. *pombe* cells. All experiment were performed in three biological replicates. CR, Congo red; Cal, Calcoflour.

Since expression of FvC5SD was found to be regulated by both temperature and pH, we performed spot dilution assay comparing the growth of *S*. *pombe* strain expressing FvC5SD with untransformed/ empty vector control cells under different temperature and pH condition. Interestingly, growth of FvC5SD expressing *S*. *pombe* transformant was comparatively more than empty vector/untransformed controls at temperature extremes of 40° C and 16° C **(Fig 5A**). However, growth of all the above mentioned strains was comparable at normal growth temperature of 30°C.

In the absence of thiamine, at pH of 5.6, which is considered normal for growth of yeast, no difference was observed in the growth of FvC5SD expressing *S*. *pombe* compared to empty vector/ untransformed control. At low pH condition (pH 3.0), created by adding oxalic acid or HCl (**Fig 5B**), FvC5SD expressing strains showed negligible growth similar to empty vector/ untransformed control under condition of thiamine repression. However, at pH 3.0, under de-repression (absence of thiamine) condition, more growth was observed in *S*. *pombe* strain expressing FvC5SD compared to controls. These observations confirm that FvC5SD expression increases tolerance of *S*. *pombe* under low pH condition.

We also compared the growth of *S*. *pombe* on cell wall inhibiting drugs like Congo red (50 μg/ml) and calcoflour white (40 μg/ml) (**Fig 5C**). FvC5SD expressing *S*. *pombe* cells showed enhanced susceptibility to the drugs effecting cell wall morphogenesis compared to empty vector/ untransformed control.

### Metabolic profiling showed enhanced ergosterol and oleic acid content in *S*. *pombe* expressing FvC5SD

**Fig 6:**
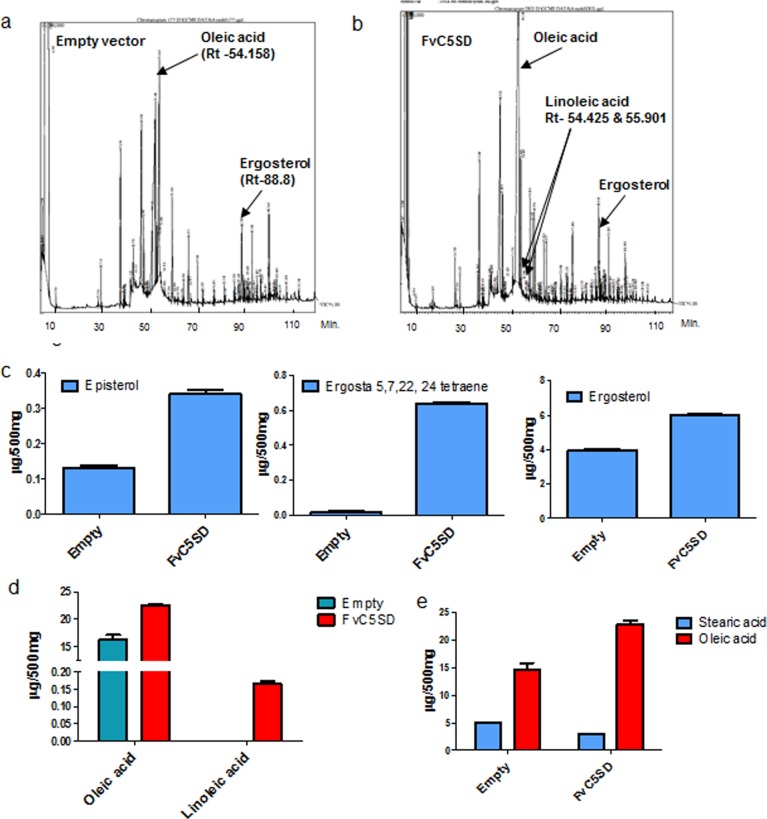
**GC-MS based comparative lipid profile of *S*. *pombe* expressing (a) Empty pSLF173 vector and (b) FvC5SD**. Peak specific to ergosterol was detected at retention time (Rt) of 88.8; oleic acid peak was detected at Rt of 54.1. Two peaks specific to linoleic acid were detected (Rt 54.4 & 55.9) only in *S*. *pombe* expressing FvC5SD. Graph comparing the levels of different type of sterols (c) episterol, ergosta 5,7, 22, 24 tetraene and ergosterol in *S*. *pombe* expressing FvC5SD and empty vector. Graph comparing level of (d) oleic acid and linoleic acid and (e) oleic acid and stearic acid in *S*. *pombe* expressing FvC5SD with empty vector control. Area of internal standard (5α-cholest7en-3β-ol) peak was used to determine the concentration of identified lipids in the given sample. Graph represents average value of three biological replicates.

Comparative metabolic profiling of total lipid extracted from *S*. *pombe* was performed by Gas chromatography- Mass spectrometry (GC-MS). The peak specific to ergosterol was detected at Rt (retention time) of 88.810 (**Fig 6A and 6B**). The peak area of internal standard was used to normalize and calculate the concentration of identified sterol. The analysis revealed that there is increase in ergosterol (ergosta-5,7, 24-trien-3β-ol) content of *S*. *pombe* strain expressing FvC5SD by **≈**1.5 folds as compared to the strain expressing empty vector (**Fig 6C**). *S*. *pombe* strain expressing FvC5SD also showed increased accumulation of other intermediates of ergosterol biosynthesis pathway such as Ergosta-5, 7, 22, 24(28)- tetraene-3β-ol (Rt: 89.7) which is the product of the enzyme C-22 desaturase (*ERG5*) that acts next to C-5Sterol desaturase (ERG3) in ergosterol biosynthesis pathway (**Fig 6C**). Episterol (ergosta-7, 24 (28)-dien-3β-ol) which is known to be immediate precursor of C-5sterol desaturase enzyme was observed to show less accumulation in FvC5SD expressing *S*. *pombe* strain compared to empty vector control, confirming enhanced C-5 Sterol desaturase activity (**Fig 6C**).

Interestingly, analysis of fatty acid profile showed that there is around 1.5 folds increase in level of monounsaturated C-18 oleic acid (detected at the Rt of 54.158) in *S*. *pombe* expressing FvC5SD (**Fig 6D)**. Besides, omega-6 polyunsaturated fatty acid, linoleic acid (two peaks at Rt of 54.425 and 55.901) was also detected in small amount in FvC5SD expressing strain which was found to be absent in control *S*. *pombe* strain. The level of C-18 saturated fatty acid i.e. octadecanoic acid or stearic acid (Rt: 54.647) in control yeast strain with empty vector was almost twice as compared to FvC5SD expressing strain. However, the ratio of oleic / stearic acid **(Fig 6E**) was found to increase in *S*. *pombe* expressing FvC5SD (oleic / stearic **≈** 9.0) compared to control (oleic / stearic **≈**3).

## Discussion

FvC5SD is an ergosterol biosynthesis enzyme of *F*. *velutipes* which was observed to be significantly up-regulated at extremes of temperature and low pH. Besides, *S*. *pombe* expressing FvC5SD showed enhanced tolerance to temperature extremes and low pH. These results show that FvC5SD play an important role under conditions of extreme temperatures and low pH stress. However, susceptibility of FvC5SD expressing *S*. *pombe* to cell wall inhibiting drugs like Congo Red and Calcoflour white increased noticeably. In order to explain the reason behind these changed properties of *S*. *pombe*, we performed total lipid profiling by GC-MS. In an earlier study, ergosterol biosynthesis genes deletion strain of *S*. *cerevisiae* (ERG2, ERG3, ERG4, ERG5, ERG6, ERG24 and ERG 28) were found to show sensitivity to lactic acid, acetic acid and HCl [57] which further points towards the role of ergosterol at low pH stress. Ergosterol being the major component of fungal plasma membranes provide permeability barrier to the cell against variety of chemical stresses including low pH condition created by adding oxalic acid/ HCl. This might be the reason behind increased expression of FvC5SD in presence of oxalic acid/ HCl. Previously, genes encoding the enzymes of lipid metabolism were observed to show altered expression in the *S*. *cerevisiae* cells subjected to different stress conditions [58]. Thus, increase in ergosterol content (1.5 folds) in FvC5SD expressing *S*. *pombe* cells could be the reason behind the enhanced tolerance to low pH.

FvC5SD is co-oriented with oxalate inducible gene OXDC with their independent promoters. Genes can be clustered together on genome in such a manner that two or more clustered genes (or transcriptional units) can possibly be regulated by common cis-elements. There is a possibility of common regulatory mechanism even in functionally and structurally unrelated genes if a regulatory element for one gene is located close to the other in a cluster. *In silico* prediction analysis of 5’ upstream region of FvC5SD revealed no known Sterol regulatory element (SRE) in their promoter which acts as binding site for transcription factors like ECM22 and UPC2. Interestingly, OXDC promoter has binding site for helix-loop-helix transcription factor UPC2 and ECM22, further pointing towards some unique kind of co-regulation. In this case possibility of bend in promoter DNA can be assumed to be one of the reasons which may lead to sharing of UPC2/ECM22 transcription factors by promoters of two genes FvC5SD and OXDC. Bending in the DNA can facilitate looping that can bring distantly bound transcription factors closer together. MADS-box proteins such as MEF2A, Rlm1p etc. are known to induce DNA bending [59]. OXDC promoter also has a RLM1 binding site (-217 position) which may favour DNA bending.

FvC5SD was cloned in *S*. *pombe* expression vector pSLF 173 which has a thiamine repressible full strength nmt1 promoter. In presence of thiamine, the promoter is repressed, thus suppressing the expression of FvC5SD and underlying effect resulting in regular colony morphology and typical filipin staining pattern (cellular sterol distribution) similar to controls (empty vector with/ without thiamine). However, when FvC5SD is expressed in medium without thiamine, *S*. *pombe* colony shows altered irregular colony morphology and punctuate staining pattern with filipin. The reason could be changed sterol composition as FvC5SD is an enzyme of sterol biosynthesis pathway.

Congo red and calcoflour white bind to the cell wall and inhibit the growth of yeast. Sensitivity to Congo red is closely related to the chitin content [60, 61]. The increased ergosterol content in FvC5SD expressing *S*. *pombe* could lead to altered activity of chitin synthase leading to sensitivity towards cell wall inhibitors.

Mechanism of temperature adaptation in an organism includes several cellular functions and components. A key role is also played by the fatty acid composition of the membrane which determine its fluidity [62]. *S*. *pombe* expressing FvC5SD showed increase in C-18 monounsaturated acid oleic acid and increased oleic acid/ stearic acid ratio compared to control cells. This is a strong reason to explain the temperature tolerance of FvC5SD expressing *S*. *pombe*. Similar results were obtained by heterologous expression of FvC5SD in tomato [32]. FvC5SD can possibly carry out non-specific desaturation of fatty acids by due to relaxed substrate affinity since conserved histidine rich motifs are integral part of catalytic core in both fatty acid desaturase and sterol desaturase. Stearic acid could act as one of the possible substrates for FvC5SD as ratio of stearic acid/oleic was also found to decrease in FvC5SD expressing *S*. *pombe*.

Performance of yeast during bioethanol production and yield is compromised by the adverse effect of ethanol accumulation on cell viability. Composition of the cellular membranes [52, 53] and presence of proper sterols and unsaturated fatty acids [54, 55] are most important factors that determine the ethanol tolerance of the organism. It has been reported earlier that oleic acid is the most effective unsaturated fatty acid that can help growing yeast cells to overcome the inhibitory effect of ethanol [63]. They reported that in ethanol rich environment, oleic acid play a critical role in maintaining membrane fluidity in. Oleic acid supplementation could restore the ability to tolerate ethanol in desaturase-deficient *S*. *cerevisiae* strains. It was hypothesized that incorporation of oleic acid into lipid membranes can lead to compensatory decrease in membrane fluidity which can counteract the fluidizing effects of ethanol, thus imparting ethanol tolerance. Similarly, ethanol tolerance of FvC5SD expressing *S*. *pombe* could be attributed to increased oleic acid content. Ergosterol content is an important factor for ethanol sensitivity of the cell. Ergosterol biosynthesis gene ERG6 was observed to complement the ethanol sensitive mutant es5 of *S*. *cerevisiae* which showed reduced ability to synthesize ergosterol [14]. Besides, Novotny et al. 1992 showed that Δ^5, 7^-Sterol-accumulating *S*. *cerevisiae* cells exhibited higher ethanol tolerance. In agreement to these data, we also observed an increased accumulation of Δ ^5, 7^-Sterol (Ergosta 5, 7, 22, 24 tetraene) in yeast expressing FvC5SD along with ergosterol [64]. This was expected as FvC5SD is known to introduce C-5 double bond into the B ring of Δ7-sterols (episterol) to yield the corresponding Δ5, 7- sterols. Hence, accumulation of these sterols could be the reasons behind increased ethanol tolerance of yeast expressing FvC5SD.

## Conclusion

Yeast has important biotechnological applications in the field of fermentation and baking throughout the history. *S*. *cerevisiae* is the most commonly used yeast for this purpose. In past, most of the research has been focused on creating improved *S*. *cerevisiae* strains that can survive extreme conditions leading to efficient production of bioethanol However, fission yeast (*Saccharomyces cerevisiae*) can also be developed as potentially useful microorganism for bioethanol production by genetic engineering strategies. FvC5SD expression in *S*. *pombe* imparts two desirable characteristics in yeast- ethanol and thermotolerance needed to make the production of biofuel ethanol more cost effective.

## Supporting information

S1 Fig Northern blot analysis to determine the expression of FvC5SD under low pH condition. 15-day-old *F*. *velutipes* mycelia was treated with HCl (added to bring down the pH of the medium to 3.0) for desired time points.
(PDF)

S2 Fig Western blot analysis to detect HA tagged FvC5SD in *S*. *pombe*. 37KDa HA tagged FvC5SD was detected using anti HA monoclonal antibody raised in mouse. 100μg of total soluble protein extract from *S*. *pombe* expressing FvC5SD or empty pSLF173 vector was separated on 12.5% SDS PAGE. M, Protein molecular weight marker.
(PDF)

S1 Table List of strains used in the study. (PDF)

S2 Table Cis regulatory elements predicted in 5’ upstream region of FvC5SD gene. (PDF)
