# Supplementary material for: Expression of C-5 sterol desaturase from an edible mushroom in fisson yeast enhances its ethanol and thermotolerance
Source: PLoS One. 2017 Mar 9;12(3):e0173381. doi: 10.1371/journal.pone.0173381 (PMC5344387; doi:10.1371/journal.pone.0173381)
Supplement: S1 Fig — 15-day-old F. velutipes mycelia was treated with HCl (added to bring down the pH of the medium to 3.0) for desired time points. (PDF) [file pone.0173381.s001.pdf]

S1 Fig

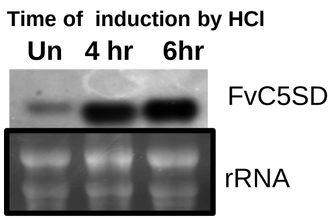

S1 Fig. **Northern blot analysis to determine the expression of FvC5SD under low pH condition**.15-day-old *F. velutipes* mycelia was treated with HCl (added to bring down the pH of the medium to 3.0) for desired time points.
