# Supplementary material for: Expression of C-5 sterol desaturase from an edible mushroom in fisson yeast enhances its ethanol and thermotolerance
Source: PLoS One. 2017 Mar 9;12(3):e0173381. doi: 10.1371/journal.pone.0173381 (PMC5344387; doi:10.1371/journal.pone.0173381)
Supplement: S2 Fig — 37KDa HA tagged FvC5SD was detected using anti HA monoclonal antibody raised in mouse. 100μg of total soluble protein extract from S. pombe expressing FvC5SD or empty pSLF173 vector was separated on 12.5% SDS PAGE. M, Protein molecular weight marker. (PDF) [file pone.0173381.s002.pdf]

**S2 Fig.**

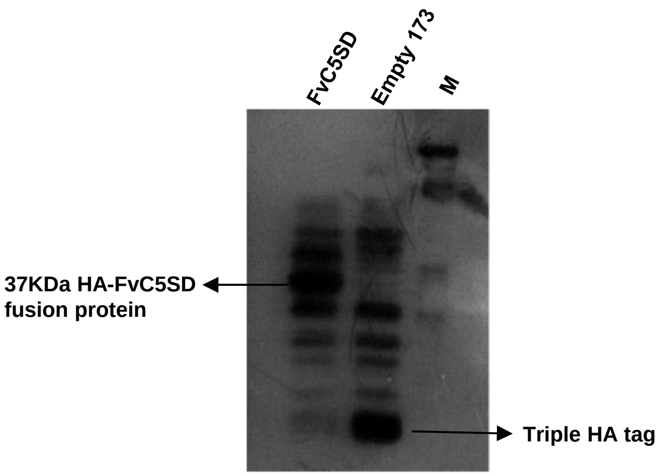


S2 Fig. **Western blot analysis to detect HA tagged FvC5SD in *S. pombe*.** 37KDa HA tagged FvC5SD was detected using anti HA monoclonal antibody raised in mouse. 100µg of total soluble protein extract from *S. pombe* expressing FvC5SD or empty pSLF173 vector was separated on 12.5% SDS PAGE. M, Protein molecular weight marker
