# Supplementary material for: Expression of C-5 sterol desaturase from an edible mushroom in fisson yeast enhances its ethanol and thermotolerance
Source: PLoS One. 2017 Mar 9;12(3):e0173381. doi: 10.1371/journal.pone.0173381 (PMC5344387; doi:10.1371/journal.pone.0173381)
Supplement: S1 Table — (PDF) [file pone.0173381.s003.pdf]

S1 Table. List of strains used in the study

Serial no.	Strain	Genotype	Source
1	*Flammulina velutipes*	ATCC-13547	ATCC
2	*Escherichia coli*	DH5α (F^- φ80*lac*ZΔM15 Δ (*lac*ZYA-*arg*F) U169 *deo*R *rec*A1 *end*A1 *hsd*R17(rk^-, mk^+) *pho*A*sup*E44 *thi*-1 *gyr*A96 *rel*A1 $λ^-$)	Lab strain
3	*Saccharomyces pombe*	BJ7468 strain (ura4-D18, leu1-32, and ade6-M216)	Lab strain
