# Supplementary material for: Expression of C-5 sterol desaturase from an edible mushroom in fisson yeast enhances its ethanol and thermotolerance
Source: PLoS One. 2017 Mar 9;12(3):e0173381. doi: 10.1371/journal.pone.0173381 (PMC5344387; doi:10.1371/journal.pone.0173381)
Supplement: S2 Table — (PDF) [file pone.0173381.s004.pdf]

S2 Table. Cis regulatory elements predicted in 5'upstream region of FvC5SD gene

| Site (Length) | Position | Score | Occurrence | Exp Value |
|---|---|---|---|---|
| Mot3-CS'(6) | 23 | 6 | 1 | 8.90e-01 |
| GCN4-ILV2'(6) | 38 | 6 | 1 | 3.08e-01 |
| rp123a-homol-D(7) | 63 | 7 | 1 | 8.78e-02 |
| GCN4-ILV1.3'(6) | 75 | 6 | 1 | 3.08e-01 |
| Tec1_CS(6) | 86 | 6 | 1 | 5.21e-01 |
| CYC1-TATA'(8) | 101 | 8 | 1 | 2.27e-02 |
| GAL1-TATA'(7) | 101 | 7 | 1 | 8.78e-02 |
| his3-Tr-TATA'(6) | 101 | 6 | 1 | 3.08e-01 |
| GCN4_CS2(10) | 127 | 10 | 1 | 8.74e-02 |
| GCN4-ILV2(6) | 133 | 6 | 1 | 3.08e-01 |
| sp-tRNA-B-box(9) | 135 | 8 | 2 | 3.07e-01 |
| MSE-S'(11) | 155 | 8 | 2 | 6.31e+00 |
| Mot3_CS(6) | 166 | 6 | 2 | 8.90e-01 |
| SWI5_consensus'(6) | 197 | 6 | 1 | 7.71e-01 |
| GCN4-HIS4.3'(6) | 211 | 6 | 1 | 3.08e-01 |
| Mot3_CS(6) | 241 | 6 | 1 | 8.90e-01 |
| Mbp1-mdscan-mot'(6) | 267 | 6 | 1 | 3.08e-01 |
| MSE-N'(10) | 287 | 8 | 1 | 5.61e-01 |
| MSE-S'(11) | 298 | 8 | 1 | 6.31e+00 |
| MSE-S(11) | 306 | 8 | 3 | 6.31e+00 |
| HSTF_consensus_'(8) | 355 | 6 | 1 | 3.07e-01 |
| MSE-S(11) | 361 | 8 | 2 | 6.31e+00 |
| DRSII_(1)'(8) | 368 | 8 | 1 | 2.27e-02 |
| Fep1_CS(6) | 369 | 6 | 2 | 5.21e-01 |
| Yox1_CS'(6) | 372 | 6 | 1 | 5.21e-01 |
| Stb5_CS3'(8) | 420 | 6 | 1 | 5.20e-01 |
| sp-tRNA-B-box(9) | 460 | 8 | 1 | 3.07e-01 |
| BAS2_site'(6) | 475 | 6 | 1 | 5.21e-01 |
| CuRE_CS2(11) | 478 | 8 | 1 | 3.86e-01 |
| Pho4_CS'(7) | 487 | 7 | 1 | 4.24e-01 |
| Tec1_CS'(6) | 492 | 6 | 1 | 5.21e-01 |
| Yap2_CA(9) | 512 | 7 | 1 | 1.67e-01 |
| AP-1_CS5'(7) | 514 | 6 | 1 | 3.08e-01 |
| AP-1_CS5'(7) | 514 | 6 | 1 | 3.08e-01 |
| BAS2_site(6) | 515 | 6 | 2 | 5.21e-01 |
| BAS2_site(6) | 518 | 6 | 1 | 5.21e-01 |
| STE12_site(6) | 520 | 6 | 1 | 3.08e-01 |
| sp-tRNA-B-box'(9) | 597 | 8 | 1 | 3.07e-01 |
| Swi5-mdscan-mot'(6) | 618 | 6 | 1 | 9.47e-01 |
| Swi5-mdscan-mot'(6) | 619 | 6 | 1 | 9.47e-01 |
| Ace2-mdscan-mot'(6) | 640 | 6 | 1 | 7.71e-01 |
| Rap1p-broad-CS'(9) | 644 | 7 | 1 | 5.20e-01 |
| DRE_CS'(10) | 659 | 9 | 1 | 8.74e-02 |
| RAD_CS'(10) | 659 | 9 | 1 | 4.47e-02 |
| PHO4_RS'(6) | 665 | 6 | 1 | 3.08e-01 |
| PHO4_RS(6) | 665 | 6 | 1 | 3.08e-01 |
| PHO4_consensus'(6) | 665 | 6 | 1 | 5.21e-01 |
| PHO4_consensus'(6) | 665 | 6 | 1 | 5.21e-01 |
| MSE-S(11) | 682 | 11 | 1 | 1.86e-01 |
| MSE-N(10) | 683 | 8 | 1 | 5.61e-01 |
| MSE-consensus(7) | 687 | 7 | 1 | 3.08e-01 |
| Fep1-CS(6) | 697 | 6 | 1 | 5.21e-01 |
| STE12-mdscan-mo'(7) | 724 | 7 | 1 | 1.68e-01 |
